# NMR Studies of Solvent-Free Ceramic Composite Polymer Electrolytes—A Brief Review 

**DOI:** 10.3390/membranes5040915

**Published:** 2015-12-14

**Authors:** Marc B. Berman, Steven G. Greenbaum

**Affiliations:** Department of Physics & Astronomy, Hunter College of the City University of New York, New York, NY 10065, USA; E-Mail: mbberman@hunter.cuny.edu

**Keywords:** polymer electrolytes, polymer ceramic composites, solid state NMR

## Abstract

Polyether-based polymer electrolytes containing ceramic inorganic oxide fillers often exhibit improved mechanical and ion transport properties compared to their filler-free counterparts. The nature of local scale interactions that give rise to these enhanced properties is explored by nuclear magnetic resonance measurements.

## 1. Introduction

The world’s need for reliable sources of energy is constantly increasing. The demand for electricity is expected to double by the year 2050 [1]. The most common source of energy comes from the burning of fossil fuels [2] which has long-term environmental and political ramifications. The need for a long-term solution may be met by renewable sources of energy. One of the most heavily studied areas in this realm is that of rechargeable batteries, which are required to store energy generated by intermittent sources (*i.e.*, solar and wind). Lithium-ion batteries have revolutionized consumer electronics and begun to make inroads in hybrid and all-electric cars. Though much development has focused on improvements in energy density, cost, and rate capability of the positive electrode, relatively little has changed in the electrolyte, which consists of a lithium salt dissolved in a mixture of carbonate solvents. Solid electrolytes have obvious safety and energy density advantages over a liquid but so far have largely failed to demonstrate sufficiently high ionic conductivity at normal operating temperatures. The recent discovery of highly conducting ceramics such as Li_10_GeP_2_S_12_ shows promise [3] in certain applications such as thin film batteries, but is challenged by the requirement of maintaining a good electrode/electrolyte interface over repeated charge and discharge. In the realm of “soft” solid electrolytes, there are gels consisting of liquid electrolytes immobilized within the gel structure and solvent-free polymer electrolytes. It is the latter that this review will highlight. Solid polymer electrolytes (SPEs) have the benefits of being flame-resistant, flexible and generally low-cost in design [4]. The research associated with lithium-ion batteries has certainly seen vast improvements over the years but can still benefit as the next generation of these types of batteries comes to fruition. Other battery technologies that could benefit from a suitable SPE are also being studied, including lithium-air and sodium-ion systems, the latter of which will be briefly addressed later. 

There are many experimental techniques that can be used to study SPEs, including electrochemical impedance spectroscopy (EIS), X-ray diffraction, neutron scattering, vibrational spectroscopy, as well as nuclear magnetic resonance (NMR) spectroscopy. NMR spectroscopy is a very useful technique because it can probe local structure as well as ionic and polymer chain and segmental dynamics [5]. NMR spectroscopy is element- (nuclei-) specific so that individual ions or functional groups can be specifically targeted. Some of the common nuclei for SEM materials studied are ^1^H, ^2^H, ^6,7^Li, ^11^B, ^13^C, ^19^F, ^23^Na, ^27^Al. The different NMR techniques used to investigate SPEs include static and magic angle spinning (MAS) experiments for local structure determination and relaxation and pulsed field gradient (PFG) diffusion measurements to study dynamic properties of materials. Of course, the maximum benefit of these kinds of measurements accrues through seeking correlations between the NMR results and parameters more directly related to battery performance such as ionic conductivity and cation transference number.

## 2. Results and Discussion

### 2.1. Polymer/Ceramic Composite Electrolytes

Poly(ethylene) oxide (PEO) is one of the most commonly studied polymers as a host material to create an effective solid polymer electrolyte [4]. Among the salts (and by no means an exhaustive list) added to PEO to form the SPE are LiClO_4_, LiI, LiCF_3_SO_3_, and LiN(CF_3_SO_2_) (often referred to in the literature as LiTFSI or LiNTf2). Despite nearly 40 years of research on PEO-salt complexes, improvements in electrical and mechanical properties for battery applications can best be described as incremental. Among early strategies to improve mechanical properties was the incorporation of ceramic particles such as Al_2_O_3_ [6], SiO_2_ [7], LiAlO_2_ [8], TiO_2_ [9] and other inorganic oxides [10]. In the course of investigating these polymer/lithium salt/ceramic composites, it was subsequently observed that the presence of the ceramic particles can also have a positive influence on ionic motion, and in favorable circumstances can even increase the Li^+^ transference number, which is the fraction of the ionic current carried by the Li^+^ ions. These effects on the ions have been attributed to ionic interaction with surface groups on the ceramic particles, and naturally such effects are markedly enhanced when using nano-sized particles in the composite [11]. Another mechanism for the enhancement of ionic conductivity is that, in many cases, the ceramic particles tend to suppress the much less conductive crystalline phases of PEO and PEO-salt complex, and, as an added benefit, the filler can also yield a more stable electrolyte/electrode interface [12,13].

Rather than offer an extensive review of the literature on the subject of ionically conductive polymer ceramic composites and NMR studies of these materials (for which several extensive reviews exist), we pose here a limited survey of examples from the labs of the authors and collaborators and those of a select few others that will serve to illustrate the main thrusts in these areas. We highlight, among other works, collaborative efforts with the laboratory of Professor Bruno Scrosati, in whose honor this volume has been compiled, as well as other notable publications from his laboratory [6].

Prior studies by the author in collaboration with Peled, Golodnitsky *et al*. [14] on PEO-Li salt complexes containing Al_2_O_3_ filler showed enhanced lithium transport in both total conductivity and cation transference number.

In fact, because it was hypothesized in this investigation that there might be more than one kind of mobile Li ion, at least one coordinated to the polyether segments and one associated with the filler particle or polymer/filler particle interfaces, a follow up study was performed on milled mixtures of LiI and Al_2_O_3_ [14]. Clear evidence of interfacial Li was observed in these mixtures using ^7^Li high resolution magic angle-spinning NMR as described later. Similar phenomena were reported some 25 years prior to this work by Liang, though without the benefit of high resolution NMR [15]. We return to this phenomenon of interfacial Li in polymer electrolyte composites later.

**Figure 1:**
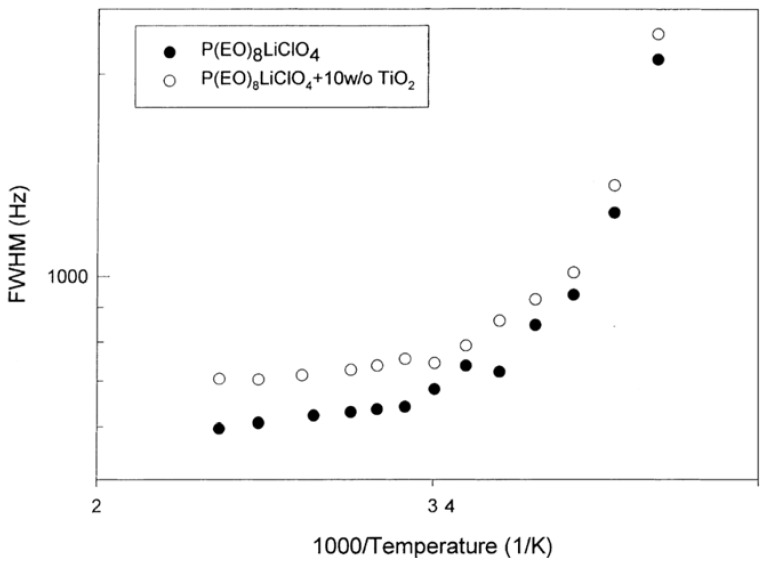
Arrhenius plot of ^7^Li NMR linewidth of Li SPE with and without TiO_2_ filler [9].

Some early evidence that the Li^+^ transport mechanism can be influenced in a fundamental way beyond that due to only reduction in the crystalline phase was provided by an investigation of TiO_2_ nanofillers in PEO_8_LiClO_4_ [9]. Figure 1 displays the ^7^Li NMR linewidth (full-width at half-maximum) of the SPE with and without the TiO_2_ filler. NMR linewidth studies provided the earliest insight into the coupling between ion transport and polymer segmental motion occurring in the amorphous phase above the glass transition temperature which usually marks the onset of motional line narrowing [5].

There is no clear distinction in motional narrowing behavior attributable to the filler and, in fact, the filler-free material exhibits a somewhat narrower resonance. However, it was determined that the ionic conductivity is enhanced by about a factor of three at 90 °C by the filler. The enhancement is about a factor of 10 at 60 °C and over two orders of magnitude at 25 °C. Though the low temperature enhancement is mainly attributable to suppression of the crystalline phase, the high temperature increase arises from additional possible transport pathways. Additional evidence for this comes from cation transference measurements performed at 90 °C, for which the obtained values were 0.20 and 0.50 in the SPE without and with filler, respectively. Furthermore, Li^+^ self-diffusion coefficients measured at 65 °C by PFG revealed an order of magnitude higher value for the filler-containing material, in line with the conductivity results.

**Figure 2:**
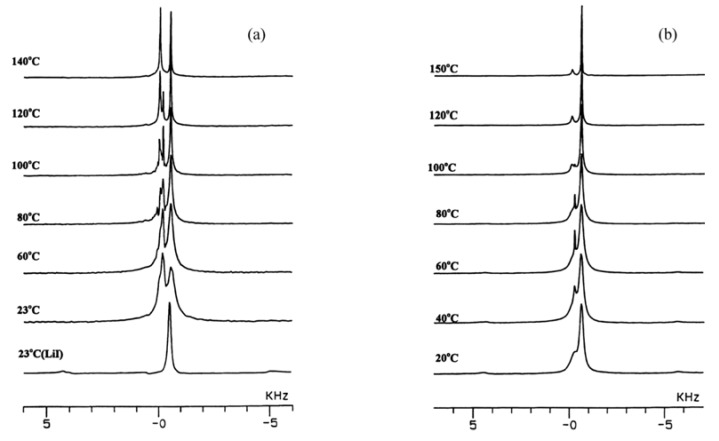
Comparison between variable-temperature ^7^Li MAS NMR spectra of LiI/Al_2_O_3_ samples: (**a**) 50/50, bottom spectrum is pure LiI reference compound; (**b**) 60/40.

Before resuming discussion of composite SPEs, we give additional evidence for the interaction between Li^+^ and ceramic filler particles even in the absence of polymer. As mentioned, this effect was first reported by Liang [15], and somewhat more recently, our group in collaboration Golodnitsky, Peled *et al*. reported on the ion conductivity enhancement of milled mixtures of LiI and Al_2_O_3_ [14]. Figure 2 shows variable-temperature high-resolution MAS solid-state ^7^Li spectra of milled mixtures of LiI and Al_2_O_3_. In addition to the bulk LiI site, there is a second resonance assigned to Li^+^ ions at the salt/ceramic interface, *i.e.*, the interfacial Li referred to earlier. The intensity of the second site is clearly greater in the sample with higher alumina content. In fact, close examination of the 50/50 composition reveals a third resolvable site at elevated temperature.

Even in the presence of polymer and resultant solvation of the ions, there is still ample opportunity for Li^+^-ceramic interactions of the kind described by Croce *et al.* [11]. These interactions produce an alternative ion transport pathway at the interface of the polymer and ceramic particle surface.

**Figure 3:**
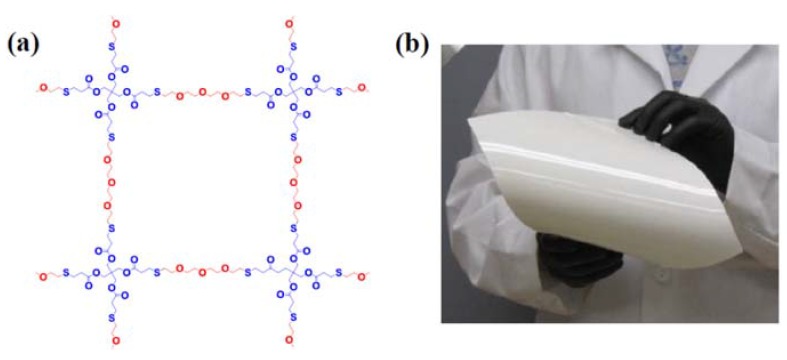
(**a**) Crosslinked PETTeDEGDVE polymer chain structure in the Al_2_O_3_/PEO membrane and (**b**) a photograph of the membrane (25 × 20 cm^2^) [15].

Another example of the benefits of inorganic oxide fillers is highlighted by the work of Park *et al*. [16]. In this particular case, a relatively large amount (30% by weight) of Al_2_O_3_ was added in order to produce a robust membrane with the ability to block dendrite formation at the anode or prevent soluble redox species at the cathode side from crossing over to the anode. The polymer structure and resultant membrane are depicted in Figure 3.

In very recent work with Golodnitsky *et al*. (Tel Aviv University), we have investigated high ceramic content PEO-LiI SPEs, where the ceramic used was LiAlO_2_, and the SPE was prepared by electrophoretic deposition (EPD) as opposed to solvent-casting [17]. The EPD method is particularly effective for restricted geometry applications such as 3-dimensional microbatteries because it allows conformal deposition of active materials.

Static ^7^Li NMR linewidth (full-width at half-maximum) measurements were performed from 25 °C up to 80 °C. As the temperature was increased, the full-width at half maximum decreased, as well as the appearance of the narrower peak which indicates a more mobile Li species. 

**Figure 4:**
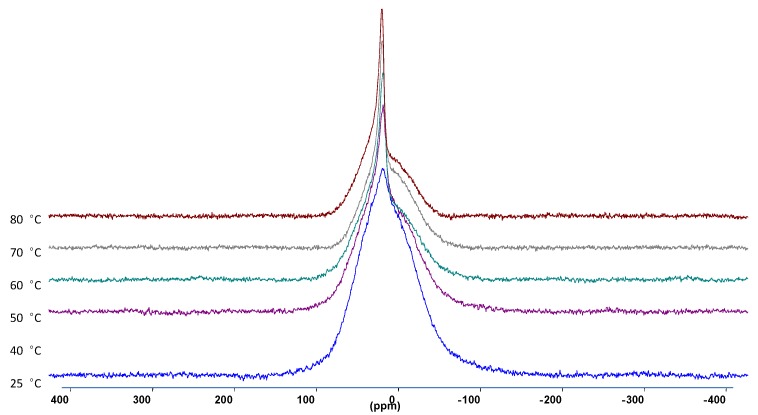
The ^7^Li NMR spectra at different temperatures of 30 wt. % LiAlO_2_ in PEO_2_LiI composite electrolyte.

**Figure 5:**
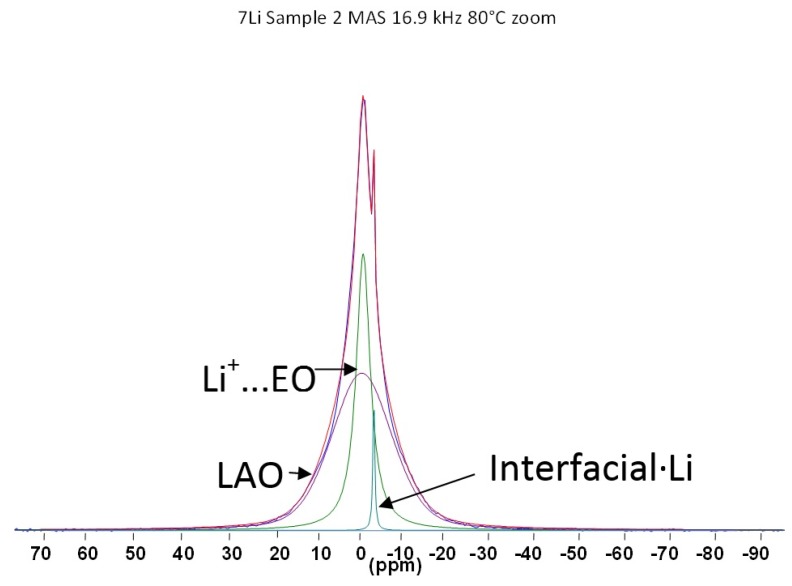
The ^7^Li NMR high resolution MAS spectrum of 30 wt. % LiAlO_2_ in PEO_2_LiI composite electrolyte at 80 °C; full spectrum plus spectral fits to components. Reproduced by permission of the Electrochemical Society.

Knowing that LiAlO_2_ itself (as opposed to the composite) yields a temperature-independent (over the range measured) linewidth, it can be assumed that the mobile species observed in the spectra correspond to Li ions either coordinated to the PEO segments or associated with the ceramic interfacial sites. In order to distinguish between these scenarios, high resolution MAS measurements were performed, as shown in Figure 5. The polymer-associated Li^+^ ions can clearly be resolved from the Li ions associated with the inorganic filler surfaces.

### 2.2. Sodium-Ion Batteries:

Recently, the first-ever vehicle powered by sodium-ion battery technology was unveiled [18]. High temperature sodium batteries were explored in the 1970s–1980s but have undergone a revival among researchers [19]. Because of their similar (to Li) electrochemical properties, such as a favorable redox potential (−2.71 for Na and −3.04 for Li) and reduced cost, sodium-based batteries operating at or near ambient temperature are receiving increasing attention [20]. Many of the materials that can be used for Li-ion batteries that have been studied over the past 25+ years are similar to that of the sodium battery [19]. One of the main hurdles to the development of these batteries is the absence of a sufficiently conductive electrolyte with the required mechanical, thermal, and electrochemical properties. 

LiTFSI mixed with PEO has been studied extensively. For sodium-based battery applications, PEO_n_:NaTFSI has been previously studied [21]; however, these films were prepared using a solvent-casting method. Recently, Moreno *et al*. [22] published their findings on a PEO_n_:NaTFSI. The addition of the nanometric-sized SiO_2_ and the use of a solvent-free hot pressing technique were chosen initially to enhance the mechanical properties of the membranes and to increase the amorphous fraction of the membranes, two of the well-documented effects discussed previously [23,24]. However, an additional benefit of the filler addition became apparent in that the chronoamperometric measurement of the Na^+^ transference number was found to increase as reported in Table 1.

**Table 1:** Transference number for PEO_20_:NaTFSI + x% SiO_2_; x = 0, 5, 10.

PEO_20_:NaTFSI + x% SiO_2_ x=	t_Na_^+^
0	0.39
5	0.51
10	0.48

**Figure 6:**
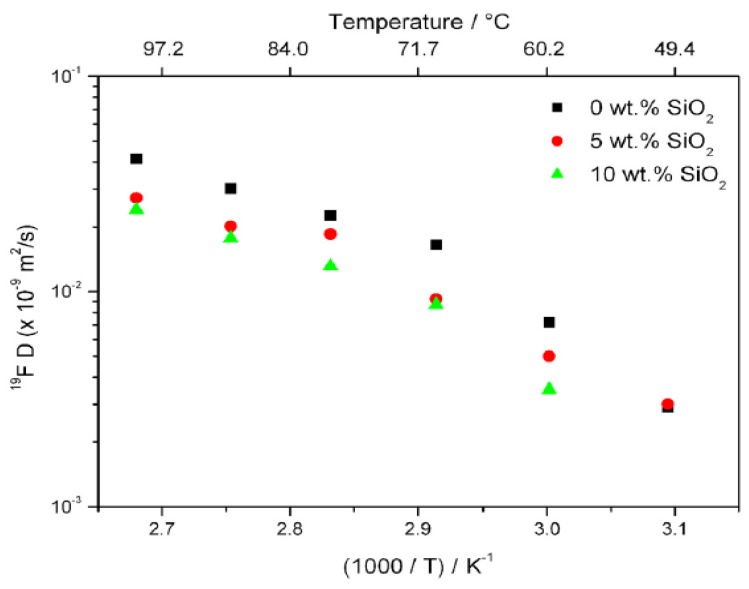
Arrhenius plot of ^19^F self-diffusion coefficients for PEO_20_:NaTFSI + x% by wt. SiO_2_ (x = 0, 5, 10).

NMR diffusion measurements were performed to gain additional insight into this phenomenon. Owing to its typically large electric quadrupole interaction and correspondingly short relaxation times, ^23^Na diffusion measurements in solids are usually not possible with normally accessible gradient strengths. Though one may not be able to measure Na diffusion, one can still determine anion diffusivity. Thus, ^19^F PFG NMR measurements showed a decrease in anion diffusivity with the addition of silica with a noticeably larger decrease in going from 0% to 5% than from 5% to 10%. The anion diffusion results are plotted in Figure 6.

It is notable that the ionic conductivity measurements showed an increase when filler was added. This is consistent with a higher Na^+^ mobility that offsets the lower TFSI^-^ anion mobility and thus an increase in the Na^+^ cation transference number, which is in agreement with the results in Table 1. 

## 3. Conclusion

Polymer electrolytes for all-solid-state batteries are promising in terms of stable, affordable, and safe storage technologies. Ceramic-polymer electrolyte composites, originally conceived to improve mechanical properties, have been shown to possess additional desirable features, including enhanced electrochemical stability against electrodes and augmented cation transference numbers. NMR is a vital investigative tool in identifying structural and mobility properties of these materials.
